# Supplementary figures and images for: Abnormal outer hair cell efferent innervation in Hoxb1-dependent sensorineural hearing loss
Source: PLoS Genet. 2023 Sep 22;19(9):e1010933. doi: 10.1371/journal.pgen.1010933 (PMC10516434; doi:10.1371/journal.pgen.1010933)

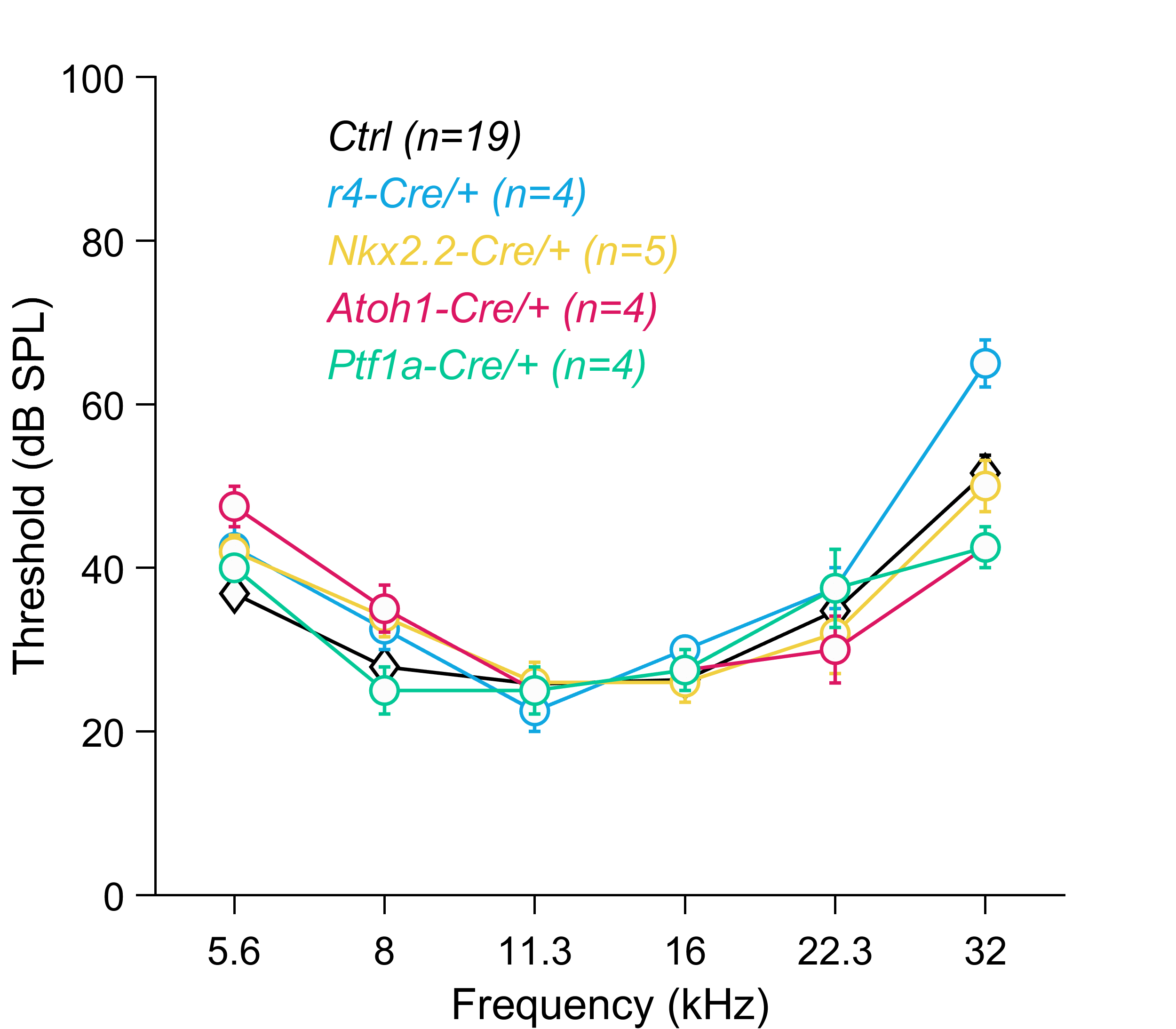

Supplement: S1 Fig — Audiograms from r4-Cre, Nkx2.2-Cre, Atoh1-Cre, Ptf1a-Cre mice. Auditory thresholds in Cre recombinase lines are comparable to Ctrl mice. Mean threshold ± SEM: 33.9 ± 0.8 dB SPL, 38.3 ± 1.4 dB SPL, 35 ± 2.5 dB SPL, 34.6 ± 0.8 dB SPL, 32.9 ± 2.3 dB SPL in Ctrl, r4-Cre, Nkx2.2-Cre, Atoh1-Cre, Ptf1a-Cre mice respectively. P = 0.27, one-way analysis of variance. See also S1 Data. (TIF) [file pgen.1010933.s001.tif]

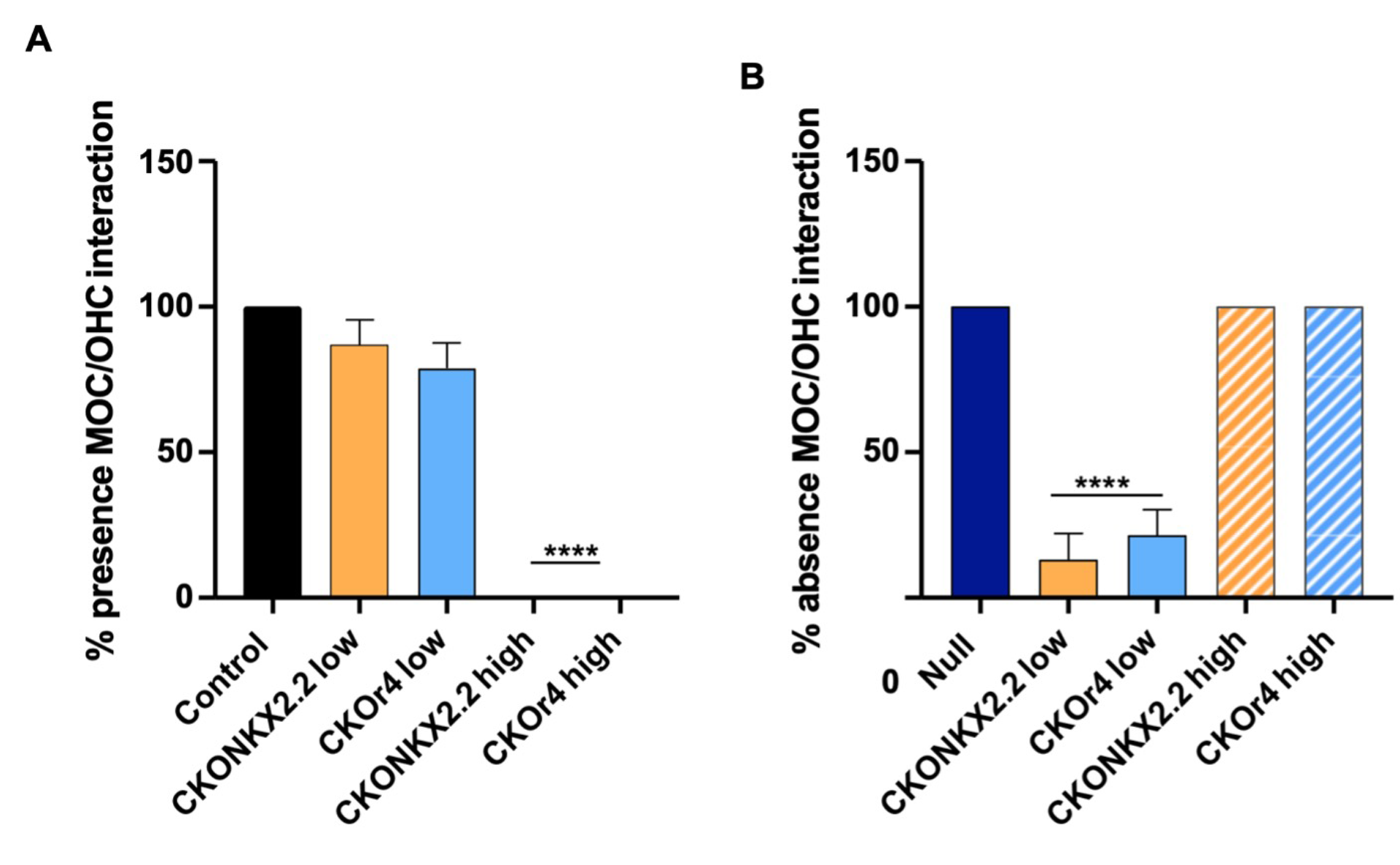

Supplement: S2 Fig — The percentage of the presence (A) of MOC/OHC interactions was quantified relative to the control group (CKONkx2.2low vs Ctrl: 88.7 ± 7.7%; P = 0.5; CKOr4low vs Ctrl: 78.6± 8.6%; P = 0.2; CKONkx2.2high vs Ctrl: 0.7 ± 0.1%; P<0.0001; CKOr4high vs Ctrl: 0 ± 0%; P<0.0001), whereas the percentage of the absence (B) of MOC/OHC interactions was quantified relative to the Null group (CKONkx2.2low vs Null: 13.0 ± 6.8%; P<0.0001; CKOr4low vs Null: 21.5 ± 7.6%; P<0.0001; CKONkx2.2high vs Null: 100 ± 6.8%; P>0.9; CKOr4high vs Null: 100 ± 6.8%; P>0.9). Data were statistically analyzed with One-Way ANOVA followed by the Bonferroni test and results are shown as mean ± SEM.**** P<0.0001. See also S1 Data. (TIF) [file pgen.1010933.s002.tif]
