# Supplementary material for: Abnormal outer hair cell efferent innervation in Hoxb1-dependent sensorineural hearing loss
Source: PLoS Genet. 2023 Sep 22;19(9):e1010933. doi: 10.1371/journal.pgen.1010933 (PMC10516434; doi:10.1371/journal.pgen.1010933)
Supplement: Attachment — Submitted filename: RESPONSES TO REVIEWERS.pdf [file pgen.1010933.s004.pdf]

RESPONSES TO REVIEWERS MS#PGENETICS-D-23-00405

Reviewer #1 :
Reviewer: Unfortunately, the data of IEE to vestibular efferents requires additional information. Given that the IEE is somewhat bilateral to the MOC the statement cited in #48 is an overstatement after cutting the OC bundle (see details of Bruce et al., 1997). Previous work showed that the loss of OHC and IHC after losing it afferents and efferents (Kersigo and Fritzsch, 2015). I strongly add the details of afferents to OHC using peripherin in P8 and adult mice (Elliott et al., 2021). I suggest tracing the IEE fibers with proper tracing to the ear.
Authors: Please, see our comments below.

Reviewer: Introduction' P3, Lin 5 cochlear cranial nerves end up in the cochlear nuclei. The distinct population is clear but does not fit to the 'vestibulocochlear' nerves (see Yang et al., 2011; Filova et al., 2022). Suggest to refer to the cochlear cranial nerve to avoid misunderstandings.
Authors: This part has now been shortened as requested by Reviewer 3. References might not be needed anymore.

Reviewer: P3, line 10 Suggest pointing out the facial MNS that migrate away (see Bruce et al., 1997).
Authors: The statement and the reference have been both added (see now page 3).

Reviewer: P4, line 5 suggest adding r2-5 for auditory nuclei
Authors: Added on page 4.

Reviewer: P4, line 15 suggest citing the work of Wong et al., 2011 that details the r4 develoment.
Authors: Reference added on page 4.

Reviewer: P6, 9 from below, suggest giving credit to Chen et al., 2012 that details very nicely the absence of r4, thank you.
Authors: Reference added on page 6.

Reviewer: P8, line 11 from below. An additional IEE are known as vestibular efferents (see Bruce et al., 1997 for details). What will happen in the absence of IEE that goes to reach out the vestibular hair cells? Will that be a parallel next review?
Authors: We thank the reviewer for these interesting questions. As previously shown, we have no vestibular efferents in the Hoxb1 null mutants (see Di Bonito et al., 2015-eNeuro PMID: 26730404 – Fig. 5). It is possible that some few cells might remain in the conditional mouse lines, but we do not think that additional analysis on vestibular efferent neurons reaching the vestibular hair cells would reinforce our results which are mainly focused on motor efferents reaching hair cells. We do NOT envisage writing an additional paper on vestibular efferents.

Reviewer: P10 The absence of IEE is shown nicely with ISH. However, my question is that all IEE are all absent in the relevant CKO mice? Perhaps a tracing of efferents could validate the suggestion as we know that even in the absence of ear neurons that IEE can redirect to the facial MNs (Ma et al., 2000).
Authors: We fully understand the reviewer's concerns. As we already showed in our previous paper in 2013 (see Fig. S5 in Di Bonito et al., 2013, PMID: 23408898) and below in Reviewer Fig. 1, almost all IEE fail to be generated in the conditional KOs at early stages. The reason is mainly because Hoxb1 plays a key role to impose an r4 identity in the developing hindbrain, and we showed by different means that in its absence r4 acquires an r3-like identity (reviewed in PMID: 23996673; PMID: 28469562). Since IEE neurons are generated in the ventral r4 Nkx2.2 domain, we expect that even in the Nkx2.2CKO, ventral r4 has changed into ventral r3-like identity, where IEE are normally not produced (which would explain the absence of Gata3). Therefore, it is this change of ventral r4 identity which prevents proper

specification of IEE the strength of our mouse models, i.e., testing the role of IEE neurons, altered by genetic means, in the amplification process of sound (differently than by lesions and/or axonal transections).

Moreover, the purpose of our study is not to follow the fate or rewiring of Hoxb1-deficient ventral r4 neurons, but to understand whether the failed interaction between MOCs and OHCs can explain the auditory threshold phenotype we describe in some mutant mice. We thus believe that the most reliable experiment to visualize OHC and MOC interactions is electron microscopy (EM) and, particularly, Transmission EM (TEM), as shown in Figs. 3 and 7. TEM was performed exactly on the same mice tested for ABR, thus strongly correlating anatomical to functional defects. The TEM analysis showed that in the CKONkx2.2 and CKOr4 mice with high thresholds, no or very few MOC/OHC interactions could be detected in all sections examined. The quantification has been added in the new Fig. S1 and S1 Data.

Regarding the tracing, we had already performed some retrograde tracing on P8 Hoxb1null and CKOr4 (previously called Hoxb1^{lateCKO}) brains in our previous paper (Di Bonito et al., 2013; PMID: 23408898 – see Fig. 6). Both genotypes have no MOC neurons crossing the midline and no obvious redirection to the facial MNs, which are also highly affected. We think that dye tracing experiments become very tricky and variable in cases of just none or very few retrogradely-labeled axons. Since the IEE-derived phenotype of the CKOr4 looks very similar to the CKONkx2.2 (see Fig. 4 in our present study), we do not expect any or very few axons to cross the midline. Again, we think that the TEM analysis, which confirms the presence or absence of OHC-MOC interactions is more adapted for our purposes.

<u>Reviewer:</u> P11 line 16 why is a _ between cochlea and MOC?
<u>Authors:</u> *Thank you for pointing out the typo*.

<u>Reviewer:</u> References:
2 should be replaced by Malmierca, 2015
3 Simmons should be replaced by Simmons et al., 2011
<u>Authors:</u> *We have added these two citations but also left the Simmons 2002 one since this review nicely describes the development of IEE across vertebrate species.*

<u>Reviewer:</u> 15 I believe the paper by Zuo et al., 1999 should be cited here
19. suggest adding the citation of Elliott et al., 2021
28. suggest adding the work of Matei et al., 2005 for complete
30 a most recent presentation by Elliott et al., 2023
31 suggesting citations such as Bruce et al., 1997
34 suggest adding the work of Karis et al., 2000 (2001)
55 suggest adding the citation of Wang et al., 2005
65 suggest adding for Wong et al., 2011 that details the feedback for Hox
<u>Authors:</u> *All these references have been added as requested by the reviewer.*

<u>Reviewer:</u> Figures
Fig. 1 Significances are needed (p; 0.05?) in B. The 32 kHz shows an effect of Hoxb1 het. What is the significance?
<u>Authors:</u> *Thank you for the question. There are no significant differences regarding threshold between wild-type and heterozygous mice, even for the 32 kHz of sound-stimulation frequency (@ 32 kHz, P-value = 0.108742, two tailed Mann-Whitney-Wilcoxon test). In the new version of Fig.1, the difference between genotypes regarding the thresholds and DPOAEs are indicated with stars (*<0.05, ***<0.001 and n.s.>0.05).*

<u>Reviewer:</u> Fig. 2 A recent publication (Elliott et al., 2023) shows the expansion of Ptf1a deletion. It would be interesting to show the effect of Ptf1 deletion in r4 that should show less expansion compared to Ptf1a deletions.

<u>Authors:</u> This is a very interesting point, but we are just using the Ptf1-Cre line to delete Hoxb1 in r4. To find out what happens if Ptf1 is deleted in r4, we would need to generate a completely new mouse line, i.e. Ptf1 floxed with b1r4-Cre, and a specific new study.

<u>Reviewer:</u> Fig. 4 A detailed tracing information was presented by Bruce et al., 1997 that clarifies the presentation of vestibular, LOC and MOCs. How many vestibular efferents develop in different CKO deletions?
<u>Authors:</u> We know from our previous paper (Di Bonito et al., 2015 eNeuro – Fig. 5) that vestibular efferents are completely lost in the Hoxb1null mutant, as demonstrated by marker expression and retrograde tracing. We have not analyzed what happens with these neurons in the other mutants, but we would expect very few of these neurons in the CKOr4 and CKONkx2.2, and no difference in the sensory-deleted mutants, since we do not inactivate Hoxb1 in IEE neurons. We do not consider that additional analysis on vestibular efferent in all mutants would reinforce our data on the interaction between MOCs and OHCs, which is the main message of our study.

<u>Reviewer:</u> Fig. 6 This information seems to compare with Maricich et al., 2009 after Hoxb1 deletions of Atoh1. Different ABRs have been described after complete deletion of Isl1 (Filova et al., 2023). How close is the ABR after different deletions?
<u>Authors:</u> The ABR from the different deletions (Hoxb1 null, CKOr4 and CKONkx2.2) did not differ between them regarding the amplitude and latencies of the ABR wave 1, 3 and 4. Comparing the mean waveforms of the ABR can be more difficult as the number of mice recorded, and the latency of the waves directly impact the average waveform. In addition, a comparison of the ABR waveforms from different labs can be tricky as the recording conditions may substantially differ (anesthesia, filters, transducers, rate of sound presentation…). We agree with the reviewer it would be interesting to know the difference that can be hidden in the ABR from the different deletions, but we would prefer not to overstate any conclusion.

<u>Reviewer:</u> Fig. 7. Nice images but will not proof the absence of MOCs. Suggest using a dye tracing to show the absence of MOCs (see Zuo et al., 1999).
<u>Authors:</u> As mentioned above, we believe that TEM is an adapted tool for identifying the innervation of MOC neurons to OHC, particularly if there are only a few neurons innervating the hair cells. We think that dye added to the cochlea would label all fibers reaching the cochlea and that the synapses between MOCs and OHCs would be difficult to detect. Moreover, and importantly, our TEM analyses were performed exactly on the same animals which went through the ABR (with high and low thresholds), supporting a strong correlation between the absence of MOC/OHCs interactions and hearing abnormalities.

<u>Reviewer:</u> Fig. 8 Suggest providing Prph type II fibers (see Elliot et al., 2021). Show the immunostaining for type II afferents and trace the LOC/MOC differentially at P8 and P40.
<u>Authors:</u> We thank the reviewer for this interesting suggestion, but we believe that this is outside the focus of our study, which is mainly to prove that MOC efferent fibers are the responsible cells for the abnormal auditory threshold observed in Hoxb1 mutants. Since Hoxb1 is not expressed in afferent fibers, we do not expect any changes in type II afferents.

Reviewer #2:
Reviewer: The manuscript is well written, figures are clear, statistics are appropriate. Some more recent literature is missing from citations (see detail below). Overall this is important work that furthers our knowledge of the role of the olivocochlear efferent system in maintaining cochlear cell health and hearing function by demonstrating that loss of olivocochlear neurons in developmental stages causes OHC degeneration.
Authors: *We thank the reviewer for acknowledging the quality and interest of our manuscript.*

Reviewer: Weaknesses of the work are that it depends on precise knockout of Hoxb1 in different cell populations using different Cre driver lines. This work does not demonstrate the precision of the knockouts, readers must reference previous works to see demonstrations of the specificity of the mouse lines. However, a helpful schematic is added to show how the cell-type specific knockouts are performed by targeting different rhombomeres in the developing nervous system. There are some variable results with the penetrance of the Cre in some of the conditional knockouts, resulting in some variable auditory function responses, but this is sufficiently discussed and analyzed in the manuscript.
Authors: *We agree with the reviewer that the schematic helps in understanding the type of deletions we have produced with the different mouse lines.*

Reviewer: Another weakness is that there is not enough evidence to demonstrate that MOC efferent terminals are consistently aberrant in the cKO animals, more detail below. Finally, the authors do not convincingly demonstrate that the effects of knockouts are limited to MOC, not LOC neurons or with contributing effects from the middle ear muscles. Contributions from these other systems may indeed contribute to pathology and are also an important finding, but the language regarding the contributions of these other systems to pathology should be more clear.
Authors: *We fully agree with the reviewer and have clarified in the discussion the reasons why we think that LOC neurons are most probably unlikely to participate in the increase of threshold levels we observed in Hoxb1 mutant mice. However, we acknowledge that LOC neurons might be involved in regulating the amplitude of the waves (pages 12-13), as suggested by lesion experiments (Darrow et al., 2006 - PMID: 17115038 and 2007 PMID: 17093118). Moreover, we cannot exclude that facial MNs might also contribute to the auditory phenotype we have described in the affected mutants. We hypothesize that the absence of the efferent reflex system (MEM and MOC) renders Hoxb1 mutant cochleae more hypersensitive to environmental sounds and that the OHCs (and consequently the cochlear amplification system) might result increasingly damaged with time determining a more pronounced progressive age-related degeneration of hearing in Hoxb1 mutant mice, as previously described (Di Bonito et al., 2013 PMID: 23408898).*

Reviewer: Major:
• Evidence of MOC axon terminal degeneration: The transmission EM images showing degeneration of MOC efferent terminals onto OHCs in the null and 'high' lines requires at a minimum more detail of how many sections were analyzed because it is possible that the examples shown are simply from a sectioning plane below OHCs that does not happen to have an MOC terminal. While this is unlikely, more description of the process of examining multiple sections through OHCs is necessary to thoroughly eliminate the possibility that MOC neurons are healthy but in other sections, or MOC neurons are unhealthy and degenerating, or small, but present, which would add additional detail to the research. At present, only a single animal is used for each condition, with only a single example image shown and little detail regarding numbers, which is simply not enough evidence. More detail is required about the experiments that were performed.
Authors: *We fully appreciate the reviewer's concern and apologize for not having clarified that the analysis was performed on multiple sections of multiple animals and not only on one animal per genotype, and that all the sections including OHCs have been carefully analyzed for the presence/absence of MOCs. We have now quantified the percentage of MOC/OHC interactions on Ctrls*

(n=3), Null (n=3), CKONkx2.2 high and low (n=6) and CKOr4 low and high (n=6). The new statistics have been added in the text and in Fig. S1 (see also S1 Data) and the numbers of samples added on page 17.

Reviewer: I would also recommend whole mount immunolabels with OC markers such as VAChT or CHAT or AChE to show a loss of MOC terminals across many more OHCs.
Authors: *We fully appreciate the reviewer's suggestion, but we are not sure that these markers would label the MOC/OHC interactions as precisely as the TEM analysis. Moreover, we deeply apologize but are unable to perform these experiments because all our mouse lines had to be cryopreserved due to the refurbishment of our animal facility.*

Reviewer: Lack of effects of cKO on LOC efferents, middle ear muscles, central neurons. In these experiments LOC efferent neurons will also be disrupted at the same time as MOC neurons. The loss of LOC neurons may also contribute to the hearing deficits observed here. Is there a way to more clearly determine whether the LOC neurons are involved, or not?
Authors: *We fully agree with the reviewer that in our experiments MOC and LOC neurons are both affected. To our knowledge, there is no way to find out whether LOC neurons alone are responsible for the Hoxb1 hearing phenotype since we would need a Cre-recombinase mouse that specifically inactivates LOC neurons. Unfortunately, we do not know the existence of such a mouse. As mentioned above, we have now better clarified in the discussion (pages 12-13) why we believe that LOC neurons are unlikely to contribute to the increased threshold levels found in mutant mice. However, we cannot exclude (and actually we propose it) that absence of LOC neurons might be instead involved in amplitude changes (see Fig. 6), as previously suggested by the group of Prof. Liberman (Darrow et al., 2006 - PMID: 17115038 and 2007 PMID: 17093118).*

Reviewer: Similar to the suggestion above, cholinergic staining in the inner spiral bundle may assess LOC innervation.
Authors: *We fully appreciate the reviewer's suggestion, but as mentioned above we do not have live animals anymore.*

Reviewer: Also, more detailed analysis of ABR wave 1 amplitudes, latencies, etc may be used to assess changes to the function of the type I SGN independent of thresholds and OHC function.
Authors: *We agree with the reviewer that a detailed analysis of ABR amplitude and latency independently of the threshold and OHC function is meaningful, notably to study the inner hair cell and/or the SGN functioning. However, to assess auditory function, we usually try to avoid repetitive high-level sound stimulation to minimize any trauma that could be elicited during the recordings. Thus, we did not probe the input-output function at a high level of stimulation (95 to 100 dB SPL), in which the activity within the cochlea is predominantly driven by the motion of the basilar membrane (a passive mechanism) and not by the OHCs.*

Reviewer: Some of the wording in the manuscript includes the possibility that LOC efferents play a role in auditory dysfunction (eg second to last sentence in the introduction) while other lines exclude LOC function (eg the final line of the introduction), and these should be clarified throughout.
Authors: *We apologize for the confusion; we have now uniformed our statements in the Introduction and Discussion.*

Reviewer: Minor:
• Some references should be added:
o Intro line 13, LOC effects – add Groff and Liberman 2003 PMID: 14615429.
o Intro 15-16 MOC effects, add Romero and Trussell 2021 PMID: 34250904 for recent evidence of CN innervation of MOC neurons, also consider that MOC neurons receive inhibition: Torres Cadenas et al 2020 PMID: 31719165

o Intro lines 17-18 and discussion page 15 – consider recent work showing that the MEM reflex is more sensitive than previously appreciated, incorporate into work as necessary – 2 works by Valero PMID: 26657094 and PMID: 29598837 – does this influence your interpretation of your results?
o Intro first paragraph, last line – consider adding more recent works from the lab of Dr. Gomez Casati
o Intro page 4 – "intermediate part of the dorsal cochlear nucleus" is not a regularly used term, what does this refer to? Granule cell domains separating VCN and DCN? Granule cell domains within deep layers of the DCN? Other?
o Discussion first paragraph, last line on page 11 – add Frank et al 2023 for neurotransmitters in efferent neurons PMID: 36876911 OK
o In multiple locations that discuss the effects of the MOC efferent system on development of central neurons Clause et al should be referenced PMID: 24853941
Authors: We have added most of the references suggested by the reviewer. Thank you very much for the suggestions. However, since we have simplified and shortened the introduction and discussion parts, as requested by reviewer 3, some references were not necessary anymore.

Reviewer: Typos:
o Intro last line, 'functionating' to 'functioning'
o Intro page 4, first full paragraph, line 6 – 'evolution' to 'evolutionarily'
o Intro page 5, 'previous to hearing functionality' to ''prior to hearing functionality'
o Discussion first paragraph – an underscore is present instead of a space before 'MOC' in the middle of the paragraph.
Authors: All these mistakes have now been corrected. We apologize to the reviewer.

Reviewer: Figure 1 legend – 'n indicates the number of cochleae recorded' should be included in the panel (D) text, not panel (E)
Authors: The legend has been changed according to the comment and the revised Figure. Thank you for noticing it.

Reviewer: The figure 1 colors look ok in a pdf, but they print poorly – consider using a different color for the Hoxb1null animals, or at least a different shade of blue. In addition, using different symbols in plots B and D for the different genotypes would aid readability.
Authors: We agree with the reviewer. Figures have been improved according to the suggestion of the reviewer.

Reviewer: The text throughout figure 2 is too small
Authors: We have now enlarged the genotypes and some of the text. Hope this is acceptable now.

Reviewer: Error bars appear to be either missing in plots 2 F and G, or are too small. I would expect them to be far larger than in plots C-E because there is obviously more spread in the data.
Authors: We agree with the reviewer, sorry for that. In plots 2 F and G, error bars were small and hidden behind marker symbols. We corrected the plots 2C-H with smaller marker symbols. Error bars are visible now.

Reviewer #3:
The study is important because HOXB1 mutations in humans cause hereditary hearing impairments. Hoxb1 global mutations in mice also cause hearing loss, as earlier work by the authors has showed. The current study extends their previous work by using conditional mouse mutagenesis to pinpoint the cellular basis of Hoxb-1-linked hearing loss. The data strongly suggest that the interactions between olivocochlear motor neurons and outer hair cells during a critical postnatal period are crucial for the establishment of the cochlear amplification of sound. Major strengths of this work include: (a) four conditional mouse mutants are characterized, (b) Thorough electrophysiological analysis and high-resolution anatomical methods are employed, (c) Side-by-side comparison of conditional and null Hoxb1 mutant mice, and (d) the wide distribution of audiogram is nicely analyzed with hierarchical classification in all mouse mutant lines.
Authors: We thank the reviewer for highlighting the strength of our study.

Reviewer: A number of weaknesses we are also identified, as described below. Most (but not all) conclusions are supported by the data.
Major issues:
1. Title: Improve title of the paper because the causal role of Hoxb1 in generating the innervation phenotype is not clearly reflected.
Authors: We have now changed the title to "Abnormal outer hair cell efferent innervation in Hoxb1-dependent sensorineural hearing loss".

Reviewer: The Introduction and Discussion can be condensed and improved.
2. Introduction: The authors are encouraged to condense and simplify the first paragraph of Introduction. Please, provide only the necessary information of cell types needed to understand the paper. Avoid the use of 10 acronyms (OHC, IHCs, VIII, CN, LL, LOC, MOC, IEE, MEM, PVCN). Are all these necessary to be mentioned for the reader to understand the paper? It seems that all the reader needs to know to understand the paper is MOC (motor neurons) and OHC (sensory neurons).
Authors: We have now simplified the first paragraph and reduced the number of acronyms, as requested. Thank you for the suggestion. It reads much better.

Reviewer: 3. Discussion: Discussion is unnecessary long and needs a lot of work to be improved. This is important. The authors are strongly encouraged to reduce it to 3-4 pages maximum (current length is 6 pages), so the key take-home messages are succinct and easy for the reader to grasp. The first 2 pages of the Discussion add new information from the literature full of acronyms and anatomical terms. This part entitled "Development and function of efferent innervation…"is not necessary, and I am not sure what the discussed nAChR studies add to this paper. These 2 pages can be substituted with a short paragraph that tells the reader what is the knowledge gap, and what is the new information that this manuscript provides. The authors are encouraged to follow this guide:
https://journals.plos.org/ploscompbiol/article?id=10.1371/journal.pcbi.1003453
Authors: We fully agree with the reviewer and apologize for the long discussion. We have now shortened it to 3 and a half pages and kept the key take-home messages. We have also clarified the different roles of the MOC and LOC neurons, and the main reasons for our conclusions on the key role of MOC neurons on the Hoxb1 auditory phenotype.

Reviewer: 4. A key message of the paper is that interactions between motor neurons and OHCs are crucial. Pre-synaptic and post-synaptic marker analysis would significantly strengthen this conclusion. Also, in the text and Abstract, the authors should make clear what do they think is the nature of this interaction. I presume it is a direct (chemical) synapse.
Authors: We agree with the reviewer that a careful analysis of pre-and post-synaptic markers on whole cochleae would strengthen our conclusion, even if we strongly think that electron microscopy is a more adapted strategy for detecting close interactions between the two cell types. We are unfortunately

unable to do the suggested experiments since we have no mice alive due to the refurbishment of our animal facility.

Reviewer: 5. Essential to the conclusions of the paper is the detailed characterization of the conditional mouse mutants. The Nkx2.2 CKO line is partially characterized. The confirmation of reduced Hoxb1 mRNA expression in Nkx2.2Cre mice in ventral r4 is convincing, but the authors conclude, based on Tbx20, that a decreased population of LOC and MOC neurons is present. However, it remains unclear whether LOC and MOC neurons are normally born but fail to differentiate and extend axons and dendrites, or they are not born at all, or born and then die. Experiments with additional MN markers (GATA3, ChAT) or cell death markers could help distinguish between these possibilities.

Authors: *Our earlier experiments on Hoxb1null and Hoxb1CKOr4 (see Fig. S5 in Di Bonito et al., 2013, PMID: 23408898) have shown specific loss of Gata3 (early marker of IEE) and Chat on E10.5 and E14.5 mutant embryos. Below the reviewer can find an additional figure on coronal sections through r4 illustrating the loss of ventral Gata3 in differentiating motor neurons (Phox2b+/Islet1+) and no particular increase of death cells in mutant embryos. At these early stages, it is very difficult to distinguish a few OC cells in conditional embryos. Since Hoxb1 is mainly implicated in impinging an r4 identity in the hindbrain (reviewed in Di Bonito et al. 2013, PMID: 23996673 and 2017 PMID: 28469562), and* IEE (including MOC and LOC) *are exclusively generated in ventral r4, the absence of Hoxb1 in ventral r4 will convert the identity of r4-specific motor neurons into a ventral r3-like identity, which will result in the loss of IEE neurons since r3 does not generate IEE neurons. Finally, since Nkx2.2 is expressed in r4 progenitors from E10.5 (see Pattyn et al., 2003, PMID: 12651891), we are quite confident that the absence of Hoxb1 exclusively in ventral r4 upon Nkx2.2-Cre activation would result in the same phenotype as Hoxb1CKOr4, as also confirmed by the absence of Hoxb1 itself in ventral r4 (Fig. 4A in this study).*

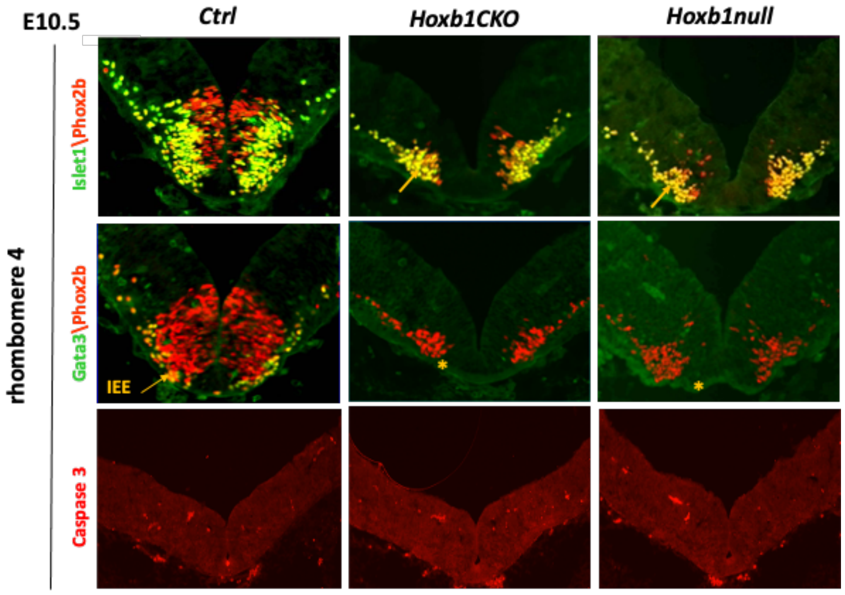

Reviewer Fig. 1: Coronal sections through r4 at E10.5 in control (Ctrl) and Hoxb1 mutants (Hoxb1CKO and Hoxb1null). Note the strong reduction of motoneuron progenitors and differentiating cells (arrows in Phox2b/Islet1), the loss (asterisks) of IEE precursors (Gata3), and no particular increase of apoptotic cells (Caspase3).

Since Gata3 staining does not label only the OC, but also other cells in adjacent zones (see Fig. S5 in Di Bonito et al., 2013, PMID: 23408898), it becomes difficult to distinguish the few residual OC neurons in conditional mice at early stages. For this reason, we preferred to use Tbx20 at late stages (P8) in this paper. While Tbx20 staining in Ctrl brains in Figure 4 nicely distinguishes LOC and MOC neurons in relation to their ventral locations, only a few cells Tbx20-expressing cells are present in the correspondent region in Hoxb1 conditional mice but not in Hoxb1null. However, Hoxb1 CKO mutants show also ectopic dorsal Tbx20 expression (see Fig. 4 in the manuscript and also Fig. 2 below), that

probably are r3-like misspecified motor neurons, in agreement with the r4 to r3-like change of identity and with the evidence that Tbx20 is also expressed the developing motor trigeminal neurons that normally derive from r3 (see also Song et al., Development, 2006, PMID: 17119020). This part has now been clarified on page 8.

We would like to clarify that this change of ventral r4 identity which prevents specification of IEE highlights the strength of our mouse models, i.e., testing the role of IEE neurons, altered by genetic means, in the amplification process of sound (differently than by lesions and/or axonal transections).

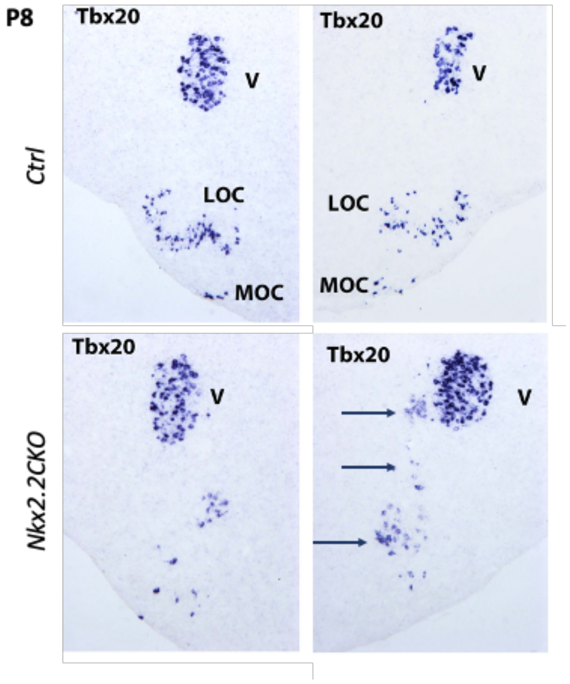

Reviewer Fig. 2: In situ hybridization of coronal sections of Ctrl and Nkx2.2CKO P8 brains showing Tbx20 staining in medial (MOC) and lateral (LOC) olivocochlear neurons as well as in the trigeminal nucleus (V). Note that in the Nkx2.2CKO, Tbx20+ cells are sparser and positioned in different ectopic locations (arrows in the bottom right image) than in control (Ctrl) brains. We do not know the exact identity of these cells, but in light of the r4 to r3-like change of identity upon Hoxb1 absence, we think that they might be r3-like misspecified motor neurons since Tbx20 is also expressed in the developing V nucleus that normally derives from r3.

Reviewer: Moreover, in Figure 4, quantifications are needed of the number of Tbx20-expressing LOC and MOC neurons, similar to what the authors did in their previous 2013 work (PMID: 23408898). The issue of what exactly happens to LOC and MOC neurons is particularly important in light of the observation that audiograms in *CKONkx2.2* line are split 50/50 in high- and low-threshold.
Authors: The P8 Tbx20 mRNA staining in Fig. 4 can vary from one embryo to the other and it becomes very difficult to quantify sparse Tbx20+ cells since they do not form a compact nucleus. These data on Tbx20 confirm earlier results in Hoxb1null and CKOr4 embryos described in our 2013 paper (Fig. S7) that were also not quantified. At this stage, it is very difficult to understand which pup will have a normal or abnormal auditory threshold. In all CKONkx2.2 pups examined we do see few or very few Tbx20+ cells with a variable pattern probably due to the timing of action or partial activity of the Cre-recombinase (see also figure above). We think that it is this timing or activity difference between Cre+ embryos that will produce high- or low-threshold groups, as we mentioned on page 9. Moreover, we have added in the new Fig. S1 a precise quantification of MOC/OHC interactions detected by TEM. We believe that this is very relevant since the mice used for electronic microscopy are the same ones tested for ABR, allowing a strict correlation between the absence or presence of MOC and hearing phenotype. Finally, by revising all the raw data that we have now added in the S1 data Excel file, we noticed some small mistakes in grouping the different genotypes. Our new analysis now confirms an even stronger phenotype among the different mouse lines, particularly in the CKONkx2.2 group in which 67% of these mice fall into the high-threshold category while only 33% belong to the low-threshold class (differently from the 50-50 reported before) (revision in Fig. 5 and on page 9).

Reviewer: 6. The manuscript mentions that OHCs degenerate (do not survive) in the the Nkx2.2 CKO line. Convincing data showing degeneration of these cells are not provided.
Authors: *The reviewer is absolutely correct. We have now changed "degeneration" to "malformation" along the text, which is what we see in the SEM pictures.*

Reviewer: 7. Data are not provided to show loss of Hoxb1 expression in sensory (inhibitory and excitatory) cochlear neurons in CKOAtoh1 and CKOPtf1a mice. This is very important in light of the absence of a phenotype in these animals (Fig. 2C-E).
Authors: *We fully understand the reviewer's concern. We have performed whole-mount ISH analysis also on early CKOAtoh1 and CKOPtf1a embryos (at the same stage as the ones shown in Fig. 4), but even after the sectioning loss of Hoxb1 expression on these embryos was not very obvious. We think this is the case because the sensory populations positive for Atoh1 and Ptf1a in r4 are very specific and most probably generated at a later time (than ventral motor neurons) and the whole-mount technique is not sensitive enough to detect them. Since the activity of the Atoh1Cre and Ptf1aCre lines on cells of the cochlear nucleus has been very well characterized in the original paper (Fujiyama et al 2009), as well as the reliability of Hoxb1floxflox mice, as demonstrated in our previous paper (Di Bonito et al 2013), we do not envisage any reason why these lines should not work.*

Reviewer: 8. Quantifications of data (as they authors did in PMID: 23408898) presented in Figure 3 and Figure 7 would strengthen the conclusions.
Authors: *We fully agree with the reviewer and have now added the quantification of the percentage of MOC/OHC interactions in Fig. S1 and individual data are listed in S1 data.*

Reviewer: Minor issues:
In Figure 1, panel A, The authors should bring the schematic (WT only) from Figure 8. This would help the non-expert reader with auditory anatomy. It would also help communicate the key take-home message of this paper, i.e., the interaction between motor neurons (MOC) and OHCs.
Authors: *We have now added a schematic in Figure 1 to help the non-expert reader to follow the different auditory steps. Figure 8 has been also updated.*

Reviewer: In Introduction, last paragraph, please clarify that null phenotype is partially (not completely) reproduced in CKOr4 and CKONkx2.2 mutants. At the end of Results section, it is also important to clarify this as well.
Authors: *We have now added "partially" to better clarify the phenotypes observed in the CKOr4 and CKONkx2.2 mutant mice.*
